# Syndrome of Irreversible Lithium-Effectuated Neurotoxicity (SILENT): A Review

**DOI:** 10.1192/j.eurpsy.2022.1850

**Published:** 2022-09-01

**Authors:** C. Fernandes Santos, R. Gomes

**Affiliations:** Hospital Garcia de Orta, Department Of Psychiatry And Mental Health, Almada, Portugal

**Keywords:** Lithium, psychopharmacology, toxicity, psychotropics

## Abstract

**Introduction:**

Lithium has a narrow therapeutic window. Frequent monitoring of both serum levels and clinical signs of toxicity is warranted because toxicity may be present even when concentrations are within the therapeutic range. Persistent neurological signs and symptoms of lithium intoxication gained clinical attention in the 1980s and were named Syndrome of Irreversible Lithium-Effectuated Neurotoxicity (SILENT).

**Objectives:**

To review the long-term neurological sequelae of lithium intoxication (SILENT) to highlight their clinical presentation, assessment, management and preventive measures.

**Methods:**

Non-systematic review of literature through search on *PubMed/MEDLINE* for publications up to 2021, following the terms *syndrome of irreversible lithium-effectuated neurotoxicity.*

**Results:**

Neurological manifestations of lithium poisoning may persist, even after effective removal of the drug – SILENT. The most frequent sequelae are cerebellum and brain stem dysfunction, extrapyramidal symptoms and dementia. They may last for weeks, months or years. Infection, dehydration, deteriorating renal function or the addition of other drugs may precipitate acute toxicity. Irreversible damage is difficult to treat. Some cases show spontaneous recovery that may be total, but in others, sequelae persist. Helpful measures include the avoidance of acute intoxications with lithium, long-term and continuous dose adjustment and serum level monitoring, stricter exclusion criteria for starting lithium, and aggressive treatment of acute neurotoxicity. Once the long-term neurologic sequelae have set in, the patient should be managed according to the impediment (physical rehabilitation, speech, cognitive training).

**Conclusions:**

It is important to raise the awareness of SILENT so that clinicians are able to avoid it. There should be a low threshold for suspecting the existence of toxicity.

**Disclosure:**

No significant relationships.

